# Multicomponent Therapeutics of Berberine Alkaloids

**DOI:** 10.1155/2013/545898

**Published:** 2013-03-24

**Authors:** Jiaoyang Luo, Dan Yan, Meihua Yang, Xiaoping Dong, Xiaohe Xiao

**Affiliations:** ^1^China Military Institute of Chinese Medicine, Integrative Medical Center of 302 Military Hospital, Beijing 100039, China; ^2^Institute of Medicinal Plant Development, Chinese Academy of Medical Sciences, Peking Union Medical College, Beijing 100193, China; ^3^College of Pharmacy, Chengdu University of Chinese Traditional Medicine, Chengdu 610075, China

## Abstract

Although berberine alkaloids (BAs) are reported to be with broad-spectrum antibacterial and antiviral activities, the interactions among BAs have not been elucidated. In the present study, methicillin-resistant *Staphylococcus aureus* (MRSA) was chosen as a model organism, and modified broth microdilution was applied for the determination of the fluorescence absorption values to calculate the anti-MRSA activity of BAs. We have initiated four steps to seek the optimal combination of BAs that are (1) determining the anti-MRSA activity of single BA, (2) investigating the two-component combination to clarify the interactions among BAs by checkerboard assay, (3) investigating the multicomponent combination to determine the optimal ratio by quadratic rotation-orthogonal combination design, and (4) *in vivo* and *in vitro* validation of the optimal combination. The results showed that the interactions among BAs are related to their concentrations. The synergetic combinations included “berberine and epiberberine,” “jatrorrhizine and palmatine” and “jatrorrhizine and coptisine”; the antagonistic combinations included “coptisine and epiberberine”. The optimal combination was berberine : coptisine : jatrorrhizine : palmatine : epiberberine = 0.702 : 0.863 : 1 : 0.491 : 0.526, and the potency of the optimal combination on cyclophosphamide-immunocompromised mouse model was better than the natural combinations of herbs containing BAs.

## 1. Introduction

To seek a multicomponent drug from traditional medicine is one of the research directions for treating MRSA infections. Rhizoma Coptidis has been used for more than two thousand years in China, and it was known as “the antibiotics in traditional Chinese medicine”. Although many studies were conducted on antimicrobial activities of berberine alkaloids (BAs) in the past few decades [1–7], few of them paid attention to the interactions among BAs. As we know, BAs are the active components of several traditional medicines, such as Rhizoma coptidis, Phellodendri Chinensis, and Berberidis Radix [8, 9], and the awareness of the interactions among BAs is of great value for the exploitation of such multicomponent drugs. 

In the present study, methicillin-resistant *Staphylococcus aureus* (MRSA) was chosen as a model organism to study the interactions among BAs. MRSA is one of the pathogenic clinically separated strains leading to high morbidity and mortality [10]. Due to its multidrug-resistant genotype, MRSA is not sensitive to the *β*-lactam antibiotics, and it is also resistant to aminoglycosides, fluoroquinolones, chloramphenicol, and macrolides [11–13], but not glycopeptides such as vancomycin and teicoplanin [14]. Single-drug treatment modalities have slowed down into bottle neck, alternatively the multi-component natural medicine might be one of the directions of the future antibacterial drug development [15]. We are expecting to clarify the synergetic and antagonistic actions and to seek the optimal combination of BAs which manifests the strongest anti-MRSA activity. We are initiating four steps to seek the optimal combination of BAs, that is, (1) determining the anti-MRSA of single BA, (2) investigating the two-component combination to clarify the interactions among BAs by checkerboard assay, (3) investigating the multi-component combination to determine the optimal ratio by quadratic rotation-orthogonal combination design, and (4) validation of the optimal combination. 

There are several methods for the compatibility study such as tubes serial dilution method and agar checkerboard dilution method. Among these methods, checkerboard microdilution is one of the commonly used assays for two-component combinations that can quickly screen the antimicrobial activity [16]. The detection sensitivity of this method could be much improved by adding the fluorescent dye of resazurin, which makes it an accurate method for determining the anti-MRSA activity of BAs. However, checkerboard microdilution is not suitable for the investigation of multi-component combinations, and hence the quadratic rotation-orthogonal combination design (QRCD) was adopted to seek optimal combinations. Compared to unifactor experiment and orthogonal design, QRCD basically retained the advantages of less experiments, simple calculation and absence of the correlation between regression coefficients. In addition, the optimal region of the concentration could be directly obtained [17, 18]. This study can help to clarify the interactions among BAs and provide a new idea for the exploitation of multi-component drugs.

## 2. Materials and Methods

### 2.1. Materials

**Figure 1:**
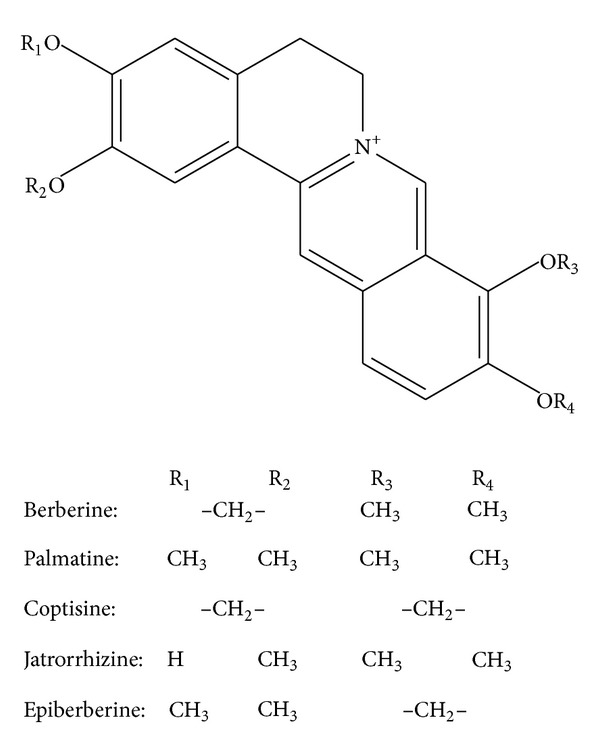
Molecular structures of berberine alkaloids.

 Rhizoma Coptidis, Phellodendri Chinensis, and Berberidis Radix were collected in the place of production. Berberine (Ber, LOT: 110713-200911) and palmatine (Pal, LOT: 110732-200907), with the purity of  ≥98%, were provided by National Institutes for Food and Drug Control. Jatrorrhizine (Jat), coptisine (Cop), and epiberberine (Epi), with the purity of  ≥97%, were purchased from Zelang Medical Technology Co., Ltd., Nanjing, China. Their structures were given in Figure 1. All other chemicals used were of analytical grade and available locally.

### 2.2. Culture

MRSA Srain (ATCC43300), *Escherichia coli* (*E. coli*, CCTCC AB91112), *Staphylococcus aureus* (*S. aureus*, CCTCC AB910393), *Shigella dysenteriae* (*S. dysenteriae*, CCTCC AB210562), *Streptococcus pneumoniae* (*S. pneumonia*, ATCC49619), and *Candida albicans* (*C. albicans*, CCTCC C091101) were provided by China Center for Type Culture Collection and were cultivated in microbiological laboratory of 302 Military Hospital of China, Beijing, China. Briefly the broth culture medium contained 10 g peptone, 6 g beef extract, and 5 g NaCl dissolving in 1000 mL deionized water (pH 7.0–7.2). Then, this culture medium was sterilized in high-pressure steam (0.1 MPa) at 121°C for 30 min. Initially, the strains were inoculated into 25 mL broth culture medium in 100 mL wide-mouthed glass bottle and incubated in a shaker for 8 h at 37°C. The rotation speed of incubator shaker was 110 rpm. The flask was enveloped with a cotton plug, so there was enough oxygen to be used by the strains. For the experiment, the bacteria were transformed and grown in cation-adjusted Mueller Hinton broth (CAMHB), which was prepared from 17.5 g casein acid hydrolysate, 3 g beef extract, and 1.5 g starch dissolving in 1000 mL distilled water (final pH 7.1–7.5 at 25°C). Then, this culture medium was sterilized in high-pressure steam (0.1 MPa) at 121°C for 30 min and stored in a refrigerator at 4°C.

### 2.3. Determination of the Activity of Single Alkaloid

Anti-MRSA activity of BAs was determined by broth microdilution according to a modified version of the Clinical and Laboratory Standards Institute (CLSI) protocol that included fluorescent determination using resazurin [19]. Resazurin is a blue dye that is nonfluorescent until it is reduced to the pink colored and highly red fluorescent resorufin. It has a large change in perceived color hue when the thickness or concentration of an observed sample increases or decreases. The fluorescence intensity of different growth stages of bacteria was determined by Synergy H1 hybrid multimode microplate reader (BioTek, Winooski, USA). The concentration of resazurin sodium salt (Sigma, Saint Louis, MO, USA) in the assay was 2.84 *μ*g/mL, and the excitation and emission wavelengths were set to 544 nm and 590 nm, respectively. Fluorescence intensity was determined 17 h after the experiment (when the fluorescence intensity peaked). The concentrations of the extracts of BAs were 2048, 1024, 512, 256, 128, 64, 32, 16, 8, and 4 *μ*g/mL for rows 1–10, respectively. Rows 11 and 12 were set as infected control (containing bacteria but no extract) and noninfected medium control (containing neither extract nor bacteria). Following fluorometric measurement, the 50% effective concentration (EC_50_) was calculated using the fluorescence intensity of rows 1–10 and the infected control.

### 2.4. Two-Component Combination Assay

**Figure 2:**
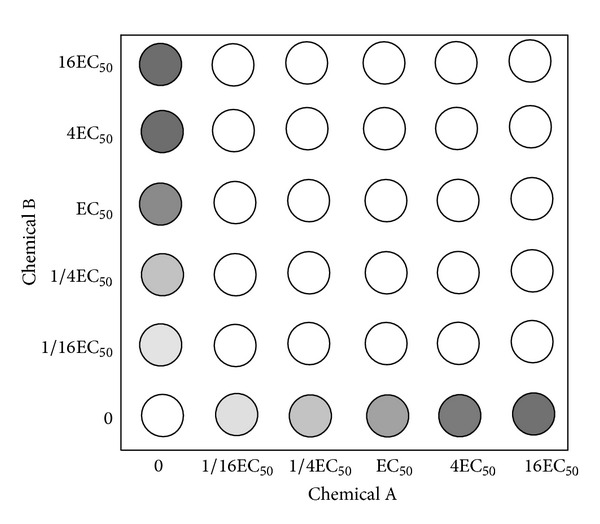
Schematic diagram of 36-point dose matrices for two-component combination assay. The five test concentrations for each compound were chosen by first determining the EC_50_ of each compound as a single agent in this assay, and then selecting 4-fold and 16-fold higher and lower concentrations.

The checkerboard assay was adopted for this assay. All 10 possible two-component combinations of these 5 active compounds were tested in duplicate in 36-point dose matrices (six doses for each compound, representing 720 data points) for their effects on the proliferation of MRSA. The five test concentrations for each compound were chosen by first determining the EC_50_ of each compound as a single agent in this assay, and then selecting 4-fold and 16-fold higher and lower concentrations, and the study is performed as Figure 2. Each combination was then scored to identify antibacterial effects that were greater than the effects of the individual components by using a resazurin proliferation assay. For each combination, we calculated the difference between the observed effect of each combination of doses and the predicted effect based on two models of additivism [20]. The highest single agent (HSA) model is the larger of the effects produced by each of the combination's single agents at the same concentrations as in the mixture. In contrast, the Bliss additivism model [21] predicts that the combined response C for two single compounds with effects A and B is C = A + B − A × B, where each effect is expressed as fractional inhibition between 0 and 1. These effect-based synergy models make no assumptions about the functional form of the dose-response curves and do not require dose-response information that lies outside the range sampled by each screening matrix. To determine whether the readout of the combination therapy is additive, synergistic, or indifferent, we calculated the fractional inhibitory concentration indices (FICI) by the checkerboard assay. The FICI represents the sum of the FICs of each drug tested, where the FIC is determined for each drug by dividing the MIC of each drug when used in combination by the MIC of each drug when used alone:

(1) FICI=(MICdrug  A  in  combinationMICdrug  A  alone)+(MICdrug  B  in  combinationMICdrug  B  alone).

### 2.5. Multicomponent Combination Assay

The QRCD was adopted to investigate the multicomponent combination of BAs. In this study, the 5 components were set as 5 factors and each factor contained 5 levels (from 100 to 500 *μ*g/mL with the interval of 100 *μ*g/mL) coded as −2, −1, 0, +1, and +2. The factor level table was designed for the experimental design of combination, and the inhibition ratios were calculated by determining the fluorescence of each combination test. The regression equation was established upon the principle of drug combination and the optimal combination could be obtained using Data Processing System (DPS, V12.01).

### 2.6. Application and Validation of the Optimal Combination

 The antimicrobial activity of the optimal combination obtained from this study was compared to three herbal medicines including Rhizoma Coptidis (the ratio of the alkaloids: Ber : Cop : Pal : Jat : Epi = 1 : 0.23 : 0.22 : 0.09 : 0.12), Phellodendri Chinensis (Ber : Pal : Jat   = 1 : 0.50 : 0.06), Berberidis Radix (Ber : Pal : Jat = 1 : 0.70 : 0.20), and geometric proportion (Ber : Cop : Pal : Jat : Epi = 1 : 1 : 1 : 1 : 1). Improved broth microdilution was used to investigate the antimicrobial activity of different BA combinations on *E. coli*, *S. aureus*, *S. dysenteriae*, *S. pneumonia,* and *C. albicans*. The inhibition ratios were calculated to evaluate the antimicrobial activity of the combinations.

### 2.7. Murine Model of Systemic Infection by Cyclophosphamide (CY)

 Before infection, mice were rendered neutropenic by i.p. injection of BAs daily for 3 consecutive days at a dosage of 100 mg/kg body weight before inoculation. Mice were then infected with 0.1 mL of inoculum of MRSA (2 × 10^6^ cfu/mL) in warmed saline (35°C) by the lateral tail vein on day 3 after pretreatment with BAs [22]. A group of mice were injected with 0.1 mL of 2 × 10^6^ cfu/mL dead MRSA suspension per mouse as negative control. In addition, a group of mice were inoculated with 0.1 ml of 2 × 10^6^ cfu/mL suspensions per mouse without advance BAs treatment as negative controls. Confirmation of infection was determined in duplicate for final isolate candidates.

Data were averaged from three experiments. All groups of mice were observed 16 days after infection. At the end point, all surviving mice were killed by CO_2_ exposure. Different tissues of mice that died during the observation period and killed survivors were removed under aseptic conditions, which were subjected to detailed necropsy examinations. Kidneys, lungs, and brains of mice were homogenized in sterile 0.9% saline. Serial dilutions of the homogenates were plated onto sabouraud dextrose agar to calculate the cfus after 48 h of incubation at 35°C. 

### 2.8. Statistical Analyses

The data obtained from QRCD were analyzed using ANOVA, and the lack of fit test of regression equation was analyzed by using DPS (DPS, V12.01). The change in bacterial density in tissues, expressed as change in log_10_ cfu for both treated and untreated animals, was reported by using descriptive statistics. Effectiveness of the combinations was undertaken with appropriate statistical tests. The mean survival time (MST) data of each group of treated mice were compared with those from untreated controls by using one-way ANOVA. Student's *t*-test was used, and the significance level was defined as *P* < 0.01.

## 3. Results and Discussion

### 3.1. Determination of the Activity of Single Alkaloid

**Figure 3:**
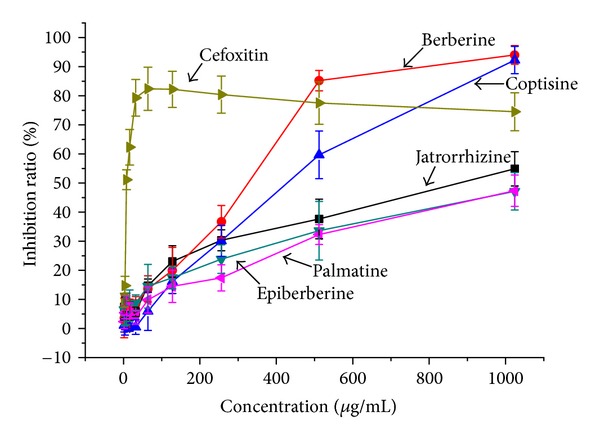
Inhibiting ratios of different concentrations of cefoxitin and five berberine alkaloids on MRSA.

The MRSA strain ATCC43300 contains *mecA* gene but no *norA* gene. In the absence of drug intervention, the fluorescence absorption reached around 40000 after 17 hours' incubation. In contrast, the fluorescence intensity was inhibited by adding the positive control cefoxitin and the alkaloids. The inhibiting effect could be calculated by the following equation: *I* = (*A* − *X*)/*A* × 100%, where *I* represents the inhibiting ratio, *A* represents the fluorescence absorption of the negative control (row 11), and *X* represents the fluorescence absorptions of alkaloids groups (rows 1–10). After that, the EC_50_ values were calculated from the *I*-concentration curves. The result showed that, among the alkaloids, berberine showed the strongest antimicrobial activity (Figure 3), and the inhibiting ratios reached 85.2% and 94.0% at the concentration of 512 *μ*g/mL and 1024 *μ*g/mL, respectively. The sequence of the activity of alkaloids was Ber > Cop > Jat > Pal > Epi. 

**Figure 4:**
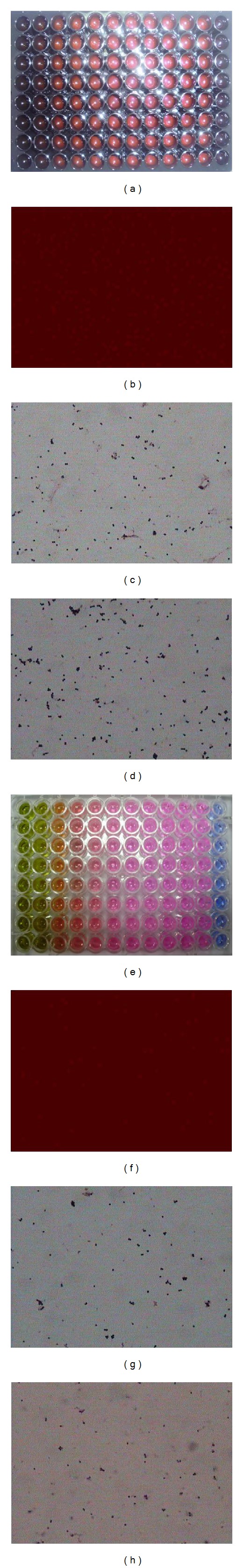
Fluorescent determination using resazurin. (a) 96-well fluorescent plates with 2-fold dilutions of Ber; (b) fluorescent image of MRSA suspension without any chemical; (c) the microphotograph of MRSA at 0 h without any chemical (stained with gram stain); (d) the microphotograph of MRSA at 24 h without any chemical; (e) 96-well translucent plates with 2-fold dilutions of Ber; (f) fluorescent image of MRSA suspension with 64 *μ*g/mL cefoxitin; (g) the microphotograph of MRSA at 24 h with 64 *μ*g/mL cefoxitin; (h) the microphotograph of MRSA at 24 h with 128 *μ*g/mL Ber.

This test adopted resazurin for fluorescent determination, and it could be found from Figure 4 that this method was reliable. When adding cefoxitin or Ber, the color of the MRSA suspension changed regularly with the increase of the concentration (Figures 4(a) and 4(b)); the fluorescence intensity at 24 h significantly decreased when adding 64 *μ*g/mL cefoxitin (Figures 4(b) and 4(f)). In addition, it showed that the density of MRSA increased significantly after being incubated for 24 h (Figures 4(c) and 4(d)); however, the colony counts significantly decreased when adding 64 *μ*g/mL cefoxitin or 128 *μ*g/mL Ber (Figures 4(g) and 4(h)).

To date, there have been some reports of drug combinations on MRSA, such as tannins and polyphenols [23], baicalein and ciprofloxacin [24], flavones and *β*-lactam antibiotics [25], usnic acid and antimicrobial agents [26], anacardic acids and methicillin [27], arbekacin and vancomycin [28], polyoxometalates and oxacillin [29], fosfomycin and arbekacin [30], gemcitabine and gentamicin [31], penicillin and *Salix babylonica* L. [32], ciprofloxacin and *Isatis tinctoria* L. [32], ceftriaxone and *Scutellaria baicalensis* G. [32], *Rosmarinus officinalis *and cefuroxime [33], and pannarin and antibiotics [34]. However, to our knowledge, there have not been any reports on the combinations of ingredients in traditional medicine. The present study showed that alkaloids had anti-MRSA activity, but the activity was relatively weak compared with antibiotics. The reason might be that the bacteria had multiple drug resistance which resulted in low absolute concentration inside the cell membrane. Bacteria have evolved numerous defenses against antimicrobial agents, and drug-resistant pathogens are on the rise [35]. A general and effective defense is conferred by ubiquitous multidrug resistance pumps (MDRs), membrane translocases that extrude structurally unrelated toxins from the cell [36–39]. 

### 3.2. Two-Component Combination Assay

**Figure 5:**
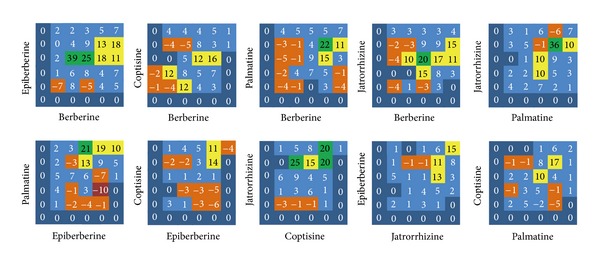
The excess over the highest single agent model, including 10 combinations of the 5 berberine alkaloids. Purple squares indicate strong antagonistic effect, orange squares indicate slight antagonistic effect, dark blue squares indicate additive effect, light blue squares indicate slight synergistic effect, brilliant yellow squares indicate strong slight synergistic effect, and green squares indicate strong synergistic effect.

**Figure 6:**
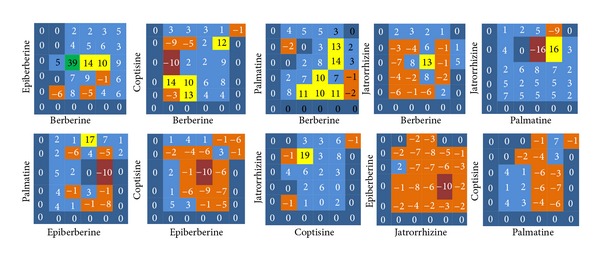
The excess over Bliss additivism model, including 10 combinations of the 5 berberine alkaloids.

 Drug interactions generally consist of synergistic, additive, and antagonistic effects [40]. The interactions among BAs are marked by 6 color gradations, among which purple indicates strong antagonistic effect, orange indicates slight antagonistic effect, dark blue indicates additive effect, light blue indicates slight synergistic effect, brilliant yellow indicates strong slight synergistic effect, and green indicates strong synergistic effect. The results showed two regularities. Firstly, the interactions among BAs were related to their concentrations, and the interactions changed along with the change of the concentrations (Figures 5 and 6). For example, while the concentration of Ber was 310.5 *μ*g/mL (EC_50_) and the concentration of Epi was 266.4 *μ*g/mL (1/4 EC_50_), 1065.4 *μ*g/mL (EC_50_), or 4261.6 *μ*g/mL (4 EC_50_), the excess over HAS > 20% and the excess over Bliss > 10%; while the concentration of Ber was 19.4 *μ*g/mL (1/16 EC_50_) and the concentration of Epi was 66.6 *μ*g/mL (1/16 EC_50_), the excess over HAS = −7% and the excess over Bliss = −6%. Secondly, different BAs showed different interactions; specifically, the combinations of Ber and Epi, Jat and Pal, Jat and Cop, Cop and Epi showed intensive synergistic effect within the test concentrations (MICs and FICIs were determined, and the result showed a synergistic effect on inhibiting CP with FICI < 0.5 or 0.5 < FICI < 1). 

By combining HAS and Bliss additivism models, it can, to a large degree, avoid false-positive results. There are a number of algorithms for quantifying synergy in the screening experiments. For example, median effect and isobolographic analyses effectively identify combinations in which one drug enhances the potency of the other drug. From the 36-point dose matrix, it was illustrated that the interactions of BAs were relevant to the proportion of each component. For example, the combination Ber & Epi showed strong synergistic effect while the proportion of Ber was 20%; the combination Pal & Epi showed obvious antagonistic effect while the proportion of Pal was 20%. The interactions among the drugs are the foundation of exploiting multicomponent drugs, and the development of such drugs should retain synergistic proportions and exclude antagonistic proportions. Following this principle, the interactions of the 10 combinations were analyzed and the optimal proportion region was obtained: Ber : Cop : Pal : Jat : Epi = 4 : 1–4 : 4–16 : 1–16 : 1–4.

### 3.3. Multicomponent Combination Assay

According to the factor level table, a total of 36 experiments were designed, and the protocol and results were shown in Table 1. The regression equation was obtained by DPS: *Y* = 0.34807 + 0.06611*X*_1_ + 0.07975*X*_2_ + 0.10040*X*_3_ + 0.04670*X*_4_ + 0.05185*X*_5_ + 0.01532*X*_1_^2^ + 0.06406*X*_2_^2^ + 0.01898*X*_3_^2^ + 0.00986*X*_4_^2^ − 0.01093*X*_5_^2^ − 0.00044*X*_1_ *X*_2_ + 0.04814*X*_1_ *X*_3_ − 0.02066*X*_1_ *X*_4_ − 0.02852*X*_1_ *X*_5_ − 0.00919*X*_2_ *X*_3_ − 0.01625*X*_2_ *X*_4_ + 0.02793*X*_2_ *X*_5_ − 0.01545*X*_3_ *X*_4_ + 0.06154*X*_3_ *X*_5_ − 0.00412*X*_4_ *X*_5_. By the analysis of variance, it showed that the lack of fit test of regression equation *F1* = MS lack of fit/MS error = 7.0009 > *F*0.01 (6, 9), *F3* = MS regression/MS error = 9.33 > *F*0.01, indicating significant. Therefore, the regression result was reliable. 

 The regression coefficient was tested for its significance, and the items that were not significant were eliminated at the level *σ* = 0.10. The equation was optimized as *Y* = 0.34807 + 0.06611*X*_1_ + 0.07975*X*_2_ + 0.10040*X*_3_ + 0.04670*X*_4_ + 0.05185*X*_5_ + 0.06406*X*_2_^2^. Analyzed by DPS, the program numbers higher than 0.41 were 1685. Meanwhile, the weighted mean and the 95% confidence interval were calculated (Table 2). Standardized the weighted mean of berberine by 1, the weighted means of other alkaloids were obtained, and the optimal ratio was obtained as follows: *X*_1_ : *X*_2_ : *X*_3_ : *X*_4_ : *X*_5_ = 0.702 : 0.863 : 1 : 0.491 : 0.526.

There have been multiple design methods, such as Central Composite Design [41], Box-Behnken Design [42], and D-optimal Design [43]. Compared with these methods, QRCD presents two features: (1) it sacrifices partial orthogonality to obtain rotatability, and it retains the merits of less test times, simple calculation, and low dependency among the regression coefficients; (2) it discards the disadvantage of lacking rotatability that cannot screen the optimal regions from predicted values. The results of this section were consistent to the results in 3.2.

### 3.4. Application and Validation

**Figure 7:**
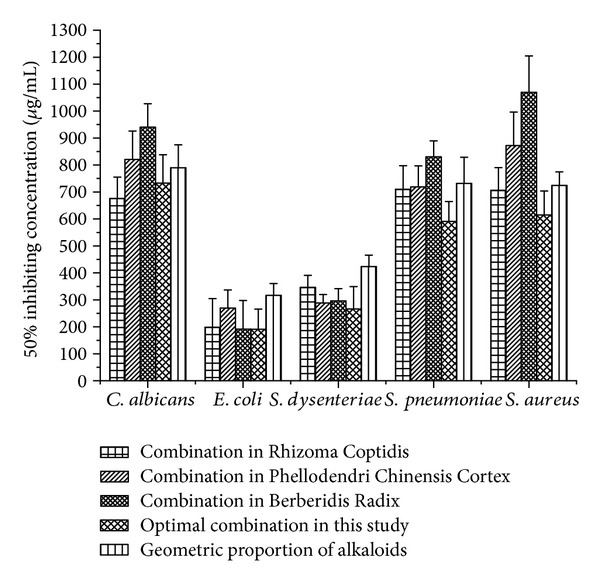
Comparison of the antimicrobial activities of different combinations of berberine alkaloids on five laboratory strains, including *E. coli*, *S. aureus*, *S. dysenteriae*, *S. pneumonia,* and *C. albicans*.

Figure 7 showed that the optimal combination in 3.3 had the strongest inhibiting effects on *E. coli*, *S. aureus*, *S. dysenteriae,* and *S. pneumonia*. Specifically, the EC_50_ of geometric proportion of alkaloids on *E. coli* was 1.81 times larger than that of the optimal combination; the EC_50_ of Berberidis Radix on *S. aureus* was 1.74 times larger than that of the optimal combination; the EC_50_ of geometric proportion of alkaloids on *S. dysenteriae* was 1.59 times larger than that of the optimal combination; the EC_50_ of Berberidis Radix on *S. pneumonia* was 1.41 times larger than that of the optimal combination. In addition, the antimicrobial activity of the optimal combination on *C. albicans* ranked second only to Rhizoma Coptidis. The results indicated that the optimal combination was not only applicable for ATCC43300, but also other laboratory strains. 

This study provided a fast and reliable way for the interaction research of chemicals, and it also laid a foundation for the development and application of multicomponent drugs. However, this study only involves *in vitro *tests, and the exploitation of multicomponent drugs still needs the validation of *in vivo *experiments. 

### 3.5. Establishment of an Antibacterial Mouse Model

**Figure 8:**
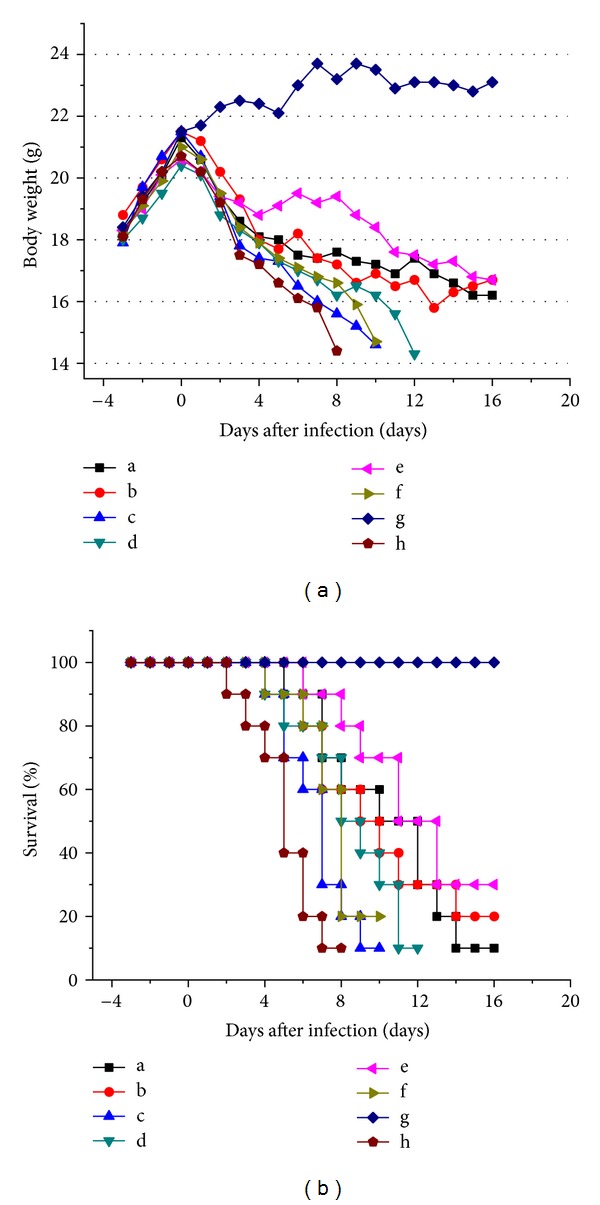
*In vivo* anti-MRSA effects of BAs combinations. (a) Body weight of ICR mice from day −3 to 16; (b) percent survival of ICR mice during the therapeutic process. (a: cefoxitin group; b: combination in Rhizoma Coptidis group; c: combination in Phellodendri Chinensis Cortex group; d: combination in Berberidis Radix group; e: optimal combination in this study group; f: geometric proportion of alkaloids group; g: negative group (noninfection mice without being given drugs); h: placebo control).

The efficacy of the synergistic pairs was tested in a living immunocompromised animal model. We developed an immunocompromised mouse model by i.p. injection of cyclophosphamide at a dosage of 100 mg/kg (body weight) once daily for 3 consecutive days to specific pathogen-free female ICR mice. To test the model with pathogens, mice were infected with 0.1 mL of MRSA suspension of 2 × 10^5^ blastospores per mouse in warmed saline (35°C) by the lateral tail vein on day 3 after pretreatment with CY. We succeeded in causing 100% mortality within 6–8 days, which was suitable for subsequent drug-evaluation experiments. To test whether the BAs combinations had synergistic effects in the CY-immunocompromised mouse model, the test compound(s) either alone or in combination were administered orally by gavage 6 h postinfection and once daily thereafter for 5 days. A control group received 0.1 mL of saline by the same route as the placebo regimens. Organs of dead and killed mice at day 16 after infection were homogenized in sterile saline, diluted, and spread onto sabouraud dextrose agar plates. Colony counts were determined after 48 h at 35°C for calculation of geometric means. Comparative efficacy assessed by changes in the bacterial density in kidney, lung, and brain tissue of the infected mice after treatment, percent survival, and MST were undertaken for test compound(s). Results obtained from Figure 8 showed that the combination in Phellodendri Chinensis Cortex and geometric proportion of alkaloids group did not have significant therapeutic effects on the body weight and percent survival of infected mice compared with placebo control (*P* > 0.05). The course of infection indicates that the MST was 5.1 ± 0.5, 6.8 ± 0.6, and 7.6 ± 0.5 for the placebo control, geometric proportion of alkaloids group, and combination in Phellodendri Chinensis Cortex group, respectively. On the contrary, the combination in Rhizoma Coptidis, cefoxitin, and optimal combination in this study group showed significant therapeutic effects (*P* < 0.01), in which the optimal combination in this study group showed the strongest anti-MRSA activity *in vivo*. The MST was 11.9 ± 1.2 for the optimal combination group. 

**Figure 9:**
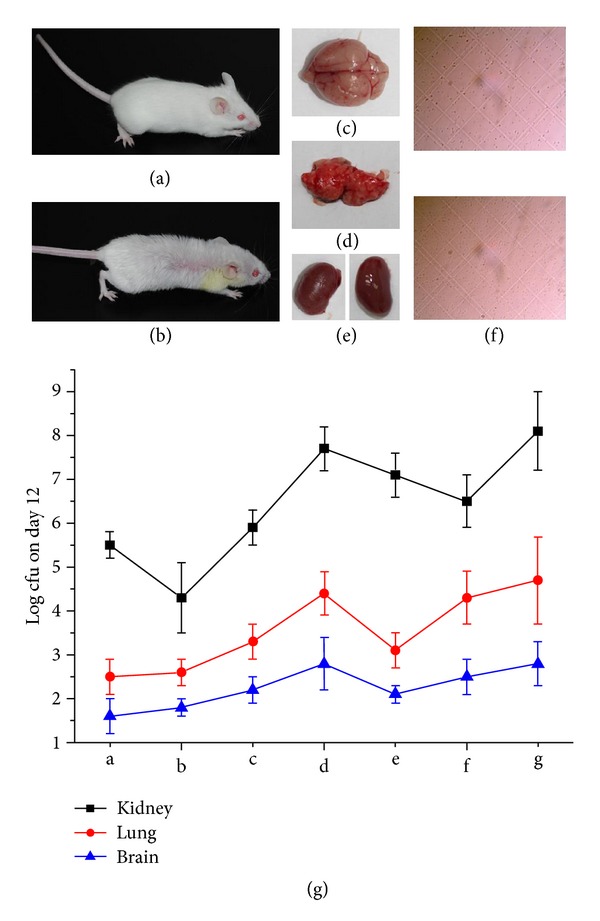
Bacterial density in kidney, lung, and brain tissues. (a) Pathogen-free female ICR mice; (b) MRSA-infected ICR mice; (c) brain tissue of died or killed mice; (d) lung tissue of died or killed mice; (e) kidney tissue of died or killed mice; (f) micrograph; (g) colony counts on day 12 (log cfu). (a: cefoxitin group; b: optimal combination in this study group; c: combination in Rhizoma Coptidis group; d: combination in Phellodendri Chinensis Cortex group; e: combination in Berberidis Radix group; f: geometric proportion of alkaloids group; g: placebo group).

After being infected by MRSA, the body weight reduced compared with placebo control, and the hair became sparse (Figures 9(a) and 9(b)). By counting the bacterial colony, it were indicated that the remaining bacteria was determined in kidney, lung, and brain tissues. Among the combinations, cefoxitin, combination in Rhizoma Coptidis, and optimal combination in this study significantly reduced the [log_10_ cfu (cell/g)] compared with placebo control. For the optimal combination, the remaining bacteria [log_10_ cfu (cell/g)] in kidney, lungs, and brain reduced dramatically from 8.1 to 4.3, 4.7 to 2.6, and 2.8 to 1.8, respectively (Figure 9(g)).

## 4. Conclusions

In summary, this study initially investigated the anti-MRSA activity of single berberine alkaloid on MRSA, and the individual EC_50_ was obtained. Thereafter, 4-fold and 16-fold higher and lower concentrations of EC_50_ were chosen for their interactions among berberine alkaloids by checkerboard assay, and the synergetic and antagonistic interactions were clarified. Next, the multi-component combination was studied by QRCD and the optimal combination for anti-MRSA activity was screened. In addition, the optimal combination was applied for its potency on other type strains, and it was demonstrated that the combination had a wide versatility. Furthermore, the BAs were tested on CY-immunocompromised mouse model to validate the therapeutic effects of the selected combinations. 

## Figures and Tables

**Table 1:** Quadratic rotation-orthogonal combination design and results.

No.	JAT	COP	BER	EPI	PAL	Inhibiting ratio (%)
1	1	1	1	1	1	90.6
2	1	1	1	−1	−1	70.0
3	1	1	−1	1	−1	50.7
4	1	1	−1	−1	1	44.1
5	1	−1	1	1	−1	66.3
6	1	−1	1	−1	1	72.9
7	1	−1	−1	1	1	30.0
8	1	−1	−1	−1	−1	32.5
9	−1	1	1	1	−1	43.0
10	−1	1	1	−1	1	70.4
11	−1	1	−1	1	1	52.3
12	−1	1	−1	−1	−1	30.4
13	−1	−1	1	1	1	61.9
14	−1	−1	1	−1	−1	26.3
15	−1	−1	−1	1	−1	37.3
16	−1	−1	−1	−1	1	16.2
17	−2	0	0	0	0	20.9
18	2	0	0	0	0	40.4
19	0	−2	0	0	0	29.3
20	0	2	0	0	0	71.0
21	0	0	−2	0	0	23.9
22	0	0	2	0	0	40.4
23	0	0	0	−2	0	17.8
24	0	0	0	2	0	39.2
25	0	0	0	0	−2	9.5
26	0	0	0	0	2	30.8
27	0	0	0	0	0	23.8
28	0	0	0	0	0	41.8
29	0	0	0	0	0	42.8
30	0	0	0	0	0	28.6
31	0	0	0	0	0	39.5
32	0	0	0	0	0	44.9
33	0	0	0	0	0	39.0
34	0	0	0	0	0	30.3
35	0	0	0	0	0	41.5
36	0	0	0	0	0	36.7

**Table 2:** Frequency distribution of the 1685 protocols with inhibiting ratio larger than 0.41.

	Weighted average	95% confidence intervals
*X*_1_ (JAT)	0.363	0.297*⋯*0.429
*X*_2_ (COP)	0.446	0.374*⋯*0.519
*X*_3_ (BER)	0.517	0.455*⋯*0.578
*X*_4_ (EPI)	0.254	0.187*⋯*0.321
*X*_5_ (PAL)	0.272	0.207*⋯*0.337
